# A systematic review about costing methodology in robotic surgery: evidence for low quality in most of the studies

**DOI:** 10.1186/s13561-018-0207-5

**Published:** 2018-09-07

**Authors:** Malene Korsholm, Jan Sørensen, Ole Mogensen, Chunsen Wu, Kamilla Karlsen, Pernille T. Jensen

**Affiliations:** 1 Department of Gynecology and Obstetrics, Faculty of Health Sciences, Odense University Hospital, Clinical Institute, University of Southern Denmark, Odense, Denmark; 2 Danish Centre for Health Economics (DaCHE), Department of Public Health, University of Southern Denmark, Odense, Denmark; 3 Healthcare Outcomes Research Centre, Royal College of Surgeons in Ireland, Dublin, Ireland; 4 Department of Pelvic Cancer, Karolinska University Hospital and Karolinska Institute, Stockholm, Sweden; 5 Clinical Institute, University of Southern Denmark, Odense, Denmark; 6 Center of Evidence-Based Medicine Odense (CEBMO), Odense University Hospital, Clinical Institute, University of Southern Denmark, Odense, Denmark; 7 Research Unit of Gynecology and Obstetrics, University of Southern Denmark, Odense University Hospital, Kløvervænget 10, 10th Floor, 5000 Odense, Denmark

**Keywords:** Economics, Robot-assisted laparoscopy, Cost analysis, Gynecologic surgery

## Abstract

**Objectives:**

The main objective of this review was to evaluate the methodological design in studies reporting resource use and costs related to robotic surgery in gynecology.

**Methods:**

Systematic searches were performed in the databases PubMed, Embase, Scopus, and The Centre for Reviews and Dissemination database for relevant studies before May 2016. The quality of the methodological design was assessed with items regarding methodology from the Consolidated Health Economic Evaluation Reporting Standards (CHEERS). The systematic review was reported according to the PRISMA guidelines.

**Results:**

Thirty-two relevant studies were included. None of the reviewed studied fully complied with the CHEERS methodological checklist. *Background and objectives*, *Target population and subgroups* and *Setting and location* were covered in sufficient details in all studies whereas the *Study perspective*, *Justification of the time horizon*, *Discount rate*, and *Estimating resources and costs* were covered in less than 50%. Most of the studies (29/32) used the health care sector perspective whereas the societal perspective was applied in three studies. The time horizon was stated in 18/32 of the studies.

**Conclusions:**

The methodological quality of studies evaluating costs of robotic surgery was low. The longest follow-up was 4 months and in general, the use of detailed cost data were lacking in most of the investigations. Key determinants, such as purchasing, maintenance costs of the robotic platform, and the use of surgical equipment, were rarely reported. If health care cost analyses lack transparency regarding cost drivers included it may not provide a true foundation for decision-making.

## Background

Previous systematic reviews comparing the cost-effectiveness of Robotic Minimally Invasive Surgery (RMIS) with other surgical modalities found higher costs associated with RMIS. However, the conclusions were based on limited evidence due to heterogeneity in study designs, methodology, and time follow-up after surgery [1–4]. In a recent review, key determinants causing higher costs of RMIS compared to open- and laparoscopic surgery were purchase and maintenance cost of the robot, surgical equipment, and additional costs related to longer operation time. The costs were, to a lesser extent, affected by the lifespan of the robotic platform and the annual number of robotic procedures [2].

The guiding principle for cost analysis is to identify the “opportunity costs”, defined as the value of the next best option [5]. Cost analyses are context specific and often limited by the availability of data. Lack of adherence to guiding principles and standards decreases transparency and quality of cost analyses [6] which may lead to wrong conclusions and decisions based on an insufficient foundation.

The aim of the present systematic review was to evaluate the methodological design employed in studies of resource use and costs related to RMIS within gynecology. We furthermore assed if the reporting quality complied with the Consolidated Health Economic Evaluation Reporting Standards (CHEERS) statement [7]. Hence, the focus was not to evaluate the cost of RMIS compared with other surgical modalities, but primarily to evaluate different methodological choices that may influence the validity of cost analyses.

## Methods

The systematic review was reported according to the Preferred Reporting Items for Systematic Review and Meta-Analyses (PRISMA) guidelines [8].

### Eligibility criteria

We included randomized controlled trials, prospective and retrospective cohorts, and case-control studies comparing the cost of hysterectomy conducted with RMIS versus laparoscopic, open, and vaginal access.

### Search

A systematic search was performed in the databases PubMed, EMBASE, Scopus, and The Centre for Reviews and Dissemination (CRD) database for relevant studies before May 2016 with assistance of a librarian. No language or date limits were imposed in the search. MK developed the PubMed search strategy with input from JS and PTJ. After the PubMed strategy was made, it was adapted to the syntax and subject headings of the other databases. Reference lists of included studies were examined for additional references [9].

### Study selection

Two authors (MK and KK) independently screened the titles and abstracts and obtained full text of all studies for the reviewing in Covidence [10]. Covidence is a component of Cochrane’s review production, a web-based systematic review tool designed to facilitate the process of screening and enables two reviewers to work efficiently through the steps of a systematic review [10]. The same two authors examined the full text articles and selected studies according to the inclusion criteria. Any inconsistency in the identification of potentially relevant papers was discussed until consensus.

### Data collection process

The CHEERS recommendations attempt to optimize reporting of health economic evaluations and consist of 24 items. We selected eight items from the CHEERS recommendations which were specifically related to the methodological design and assessed important for the reporting of costs [7]. The methodological Items included: 1) Background and objectives, 2) Target population and subgroups, 3) Setting and location, 4) Study perspective, 5) Comparators, 6) Time horizon, 7) Discount rate, 8) Estimating resources and costs. Further, we included items related to cost aspects associated to the robotic platform. The chosen items were translated into 12 questions, against which each study was assessed and marked either “yes”, “partly”, and “not available” and specification of the time follow-up after surgery (Table 1). MK and KK evaluated the quality of the methodological design in all studies independently [7]. The same authors examined their findings and any disagreement was discussed until consensus.

**Table 1:** Questions from CHEERS checklist

Section/items	Items No	Questions
Introduction		
Background and objectives	3	Was an explicit statement of the broader context provided for the study?
		Were the study questions and its relevance for health policy or practice decisions presented?
Methods		
Target population and subgroups	4	Were the analyzed characteristics of the base case population and subgroups described, including why they were chosen?
Setting and location	5	Were relevant aspects of the system(s) stated in which the decision(s) needed to be made?
Study perspective	6	Was the perspective of the study described and related to the costs being evaluated?
Comparators	7	Were the compared interventions or strategies describe and was it stated why they were chosen?
Time horizon	8	What was the time horizon?
		Was the time horizon justified?
Discount rate	9	If relevant, which discount rate was applied?^a^
Estimating resources and costs	13	Was micro-costing applied?
		Was the costs of purchasing the RMIS platform included?
		Were the maintainance costs of RMIS reported (incl. depreciation and number of procedures)?

^a^NA: If time horizon is not more than a year

## Results

### Study selection

**Fig. 1:**
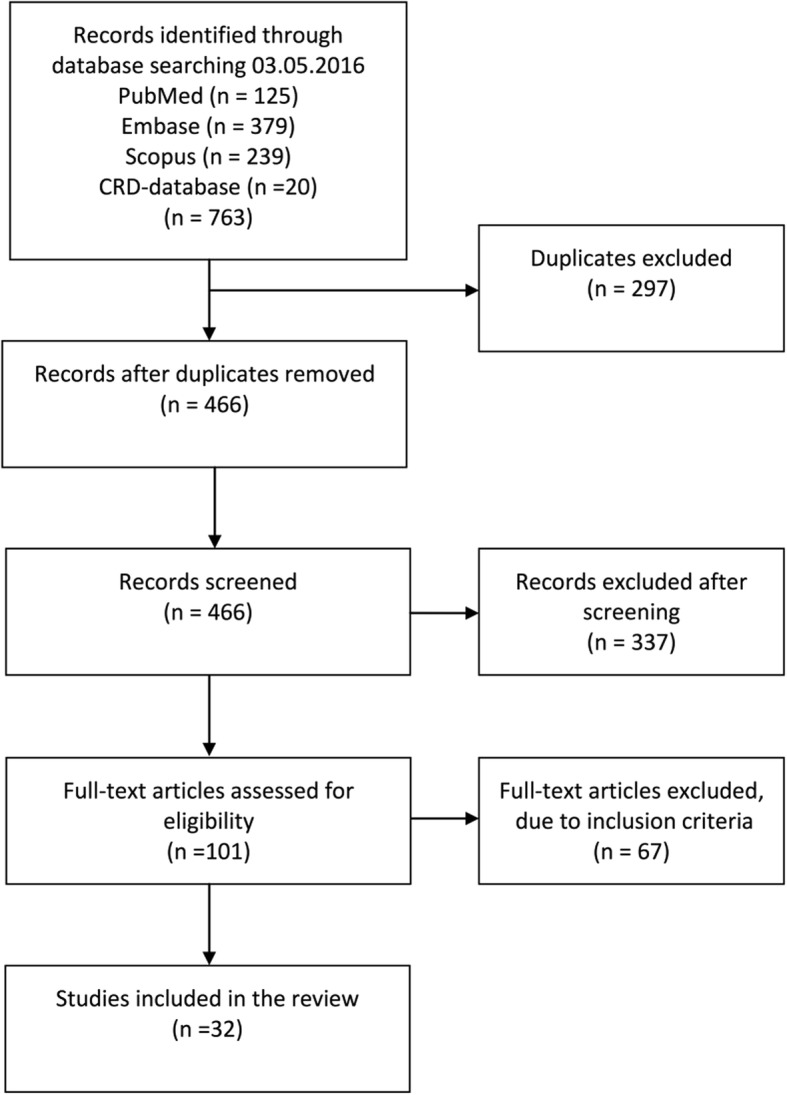
PRISMA flow chart for selection of included studies

We identified 763 references of which 32 met the inclusion criteria. The search, screening and exclusions are given in Fig. 1.

### Study characteristics

The cost studies (Table 2) were published from 2008 to 2016 and originated from the U.S. (*n* = 21), Europe (*n* = 9), and Canada (*n* = 2). In three studies, the cost analyses were conducted within the framework of a cost-minimization analysis [11, 12], one study used a cost-benefit framework [13] and one study used activity based costing [14]. The remaining studies reported on costing analyses.

**Table 2:** Characteristics of included studies

First author, country and year	Women (N)	Year of inclusion	Design	Economic evaluation	Data collection	Level
Bogani, G. (2016) U.S. [26]	251	2007–2012	Retrospective	Cost study	Chart review	Individual
Brooks, RA. (2016) U.S. [27]	6560	2010	Retrospective	Cost study	National database	Not individual
Fader, AN. (2016) U.S. [23]	32,560	2007–2011	Retrospective	Cost study	National database	Not individual
Hachem, LE. (2016) U.S. [9]	92	2013–2014	Retrospective	Cost study	Chart review	Individual
Winter, ML. (2015) U.S. [20]	183	2013	Retrospective	Cost study	Chart review	Individual
Chan, JK. (2015) U.S. [28]	1087	2011	Retrospective	Cost study	National database	Not individual
Herling, SF. (2015) Denmark [14]	360	2006–2014	Retrospective	Activity-based cost analysis	Chart review and national database	Individual
Eklind, S. (2014) Sweden [16]	88	2010–2012	Prospective	Cost study	Prospective	Individual
Shepherd, JP. (2014) U.S. [29]	4871	2011–2013	Retrospective	Cost study	Local database	Not individual
Teljeur, C. (2014) Ireland [12]	NA	NA	Retrospective	Cost-minimization analysis	Local databases, review of the literature	Not individual
Wright, JD. (2014) U.S. [30]	180,230	2006–2012	Retrospective	Cost study	Chart review	Not individual
Woelk, JL. (2014) U.S. [31]	892	2007–2009	Retrospective	Cost study	Local database and chart review	Individual
Lönnerfors, C. (2014) Sweden [17]	122	2010–2013	RCT	Cost study	Prospective	Individual
Reynisson, P. (2013) Sweden [18]	231	Jan. 2001-Feb. 2012	Retrospective	Cost study	Chart review	Individual
Desille-Gbaguidi, H. (2013) France [15]	57	2008–2011	Retrospective	Cost study	Chart review	Individual
Rosero, EB. (2013) U.S. [32]	804,551	2009–2010	Retrospective	Cost study	National database	Not individual
Wright, JD. (2013) U.S. [33]	264,758	2007–2010	Retrospective	Cost study	National database	Not individual
Turunen, H. (2013) Finland [38]	217	2009–2013	Retrospective	Cost study	Chart review	Individual
Yu, X. (2013) U.S. [39]	2247	2008–2009	Retrospective	Cost study	Local database	Not individual
Coronado, PJ. (2012) Spain [13]	347	2003–2011	Prospective and retrospective	Cost-benefit	Chart review	Individual
Wright, KN. (2012) U.S. [48]	688	2009	Retrospective	NA	Chart review	Individual
Wright, JD. (2012) U.S. [34]	2464	2008–2010	Retrospective	NA	National database	Not individual
Scribner, DR. (2012), U.S. [21]	76	2005–2009	Retrospective	NA	Chart review	Individual
Lau, S. (2012) Canada [19]	303	2003–2010	Prospective and retrospective	NA	Prospective collection and chart review	Individual
Venkat, P. (2012) U.S. [25]	54	2008–2010	Retrospective	NA	Chart review	Individual
Shah, N. (2011) U.S. [24]	234	2009	Retrospective	Cost-minimization analysis	Chart review	Individual
Jonsdottir, GM. (2011) U.S. [35]	2133	2006–2009	Retrospective	NA	Chart review	Individual
Holtz, DO. (2010) U.S. [40]	33	2005–2006	Retrospective	NA	Chart review	Individual
Pasic, RP. (2010) U.S. [36]	36,188	2007–2008	Retrospective	NA	National database	Not individual
Halliday, D. (2010) Canada [22]	40	2003–2009	Prospective and retrospective	NA	Prospective collection and chart review	Individual
Sarlos, D. (2010) Switzerland [41]	80	2007–2009	Prospective and retrospective	NA	Prospective collection and chart review	Individual
Bell, MC. (2008) U.S. [37]	110	2000–2007	Retrospective	NA	Chart review	Individual

### Results of individual studies

A visual presentation of the CHEERS criteria and the ranking of the quality assessment are shown in Table 3. All 12 questions from the CHEERS recommendations were marked either “yes”, “partly”, and “not available” for each study. A “yes” was given 1 point, “partly” a ½ point and “not available” none points.

**Table 3:**
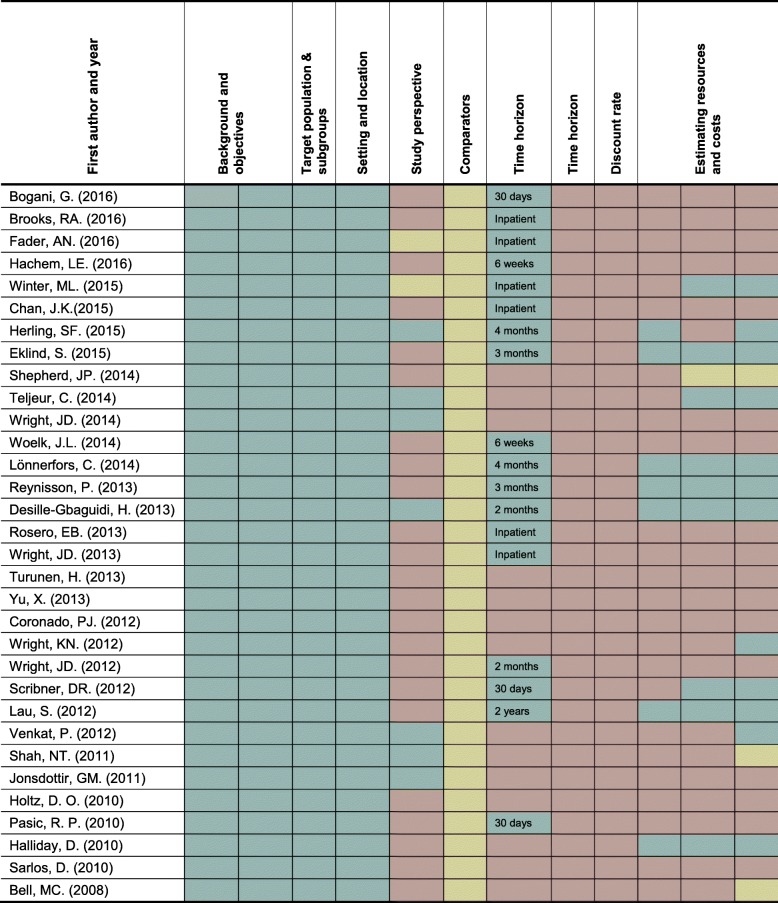
Results from CHEERS checklist.

Reported,  Partly reported,  Not reported

None of the studies reported all 12 questions; one study (3%) was given 9.5 point [15], six studies (18.8%) 8–8.5 points [14, 16–20], three studies (9.4%) 7.5 point [12, 21, 22], three studies (9.4%) 6–6.5 points [23–25], fourteen studies (43.8%) 5–5.5 points [9, 26–37] and five studies (12.5%) 4.5 point [13, 38–41].

### Results from the 12 questions

“Background and objectives”, “Target population and subgroups” and “Setting and location” were described in all studies. Comparators were partly described in most studies (30/32) but were fully reported in two studies only. The *study perspective* was clearly stated in seven studies but was not mentioned in 23 studies and only partly mentioned in two studies. Most of the studies (*n* = 29) used the health care sector perspective whereas the societal perspective was applied in three studies only [24, 35, 37]. The societal perspective included return to work/normal daily activity and estimated lost wages [24, 35, 37]. The time horizon was stated in 18/32 studies but none provided a reason for their choice of time horizon. Most studies reported from the inpatient stay (*n* = 6) and the time horizon ranged from inpatient stay to 4 months after discharge. One study measured the cancer recurrence rate 2 years after surgery but did not report the cost of the recurrence [19].

Seven studies estimated *Resources and costs* by micro-costing (collection of detailed data on resources used to assess costs of an activity) reporting detailed data from a single hospital. The majority of cost items derived from surgery and included the purchase and maintenance costs of the robotic platform with depreciation and yearly number of procedures [14–19, 22]. One of the seven studies combined micro-costing with gross-costing using hospital charge data but did not include detailed data on resource use [14]. The discount rate was not reported in any of the studies due to the short time horizon applied. The depreciation period of the robotic lifespan ranged from five to 10 years in 8/32 of the studies and [12, 14–16, 18, 19, 22, 37] 7 years was most frequently used (*n* = 5). The number of annual robotic procedures ranged from 234 to 400 and was reported in five studies [12, 14, 16–18]. The service agreement costs of 10% of the purchasing prize of the robot were reported in five studies only [12, 15, 19, 22, 37].

**Fig. 2:**
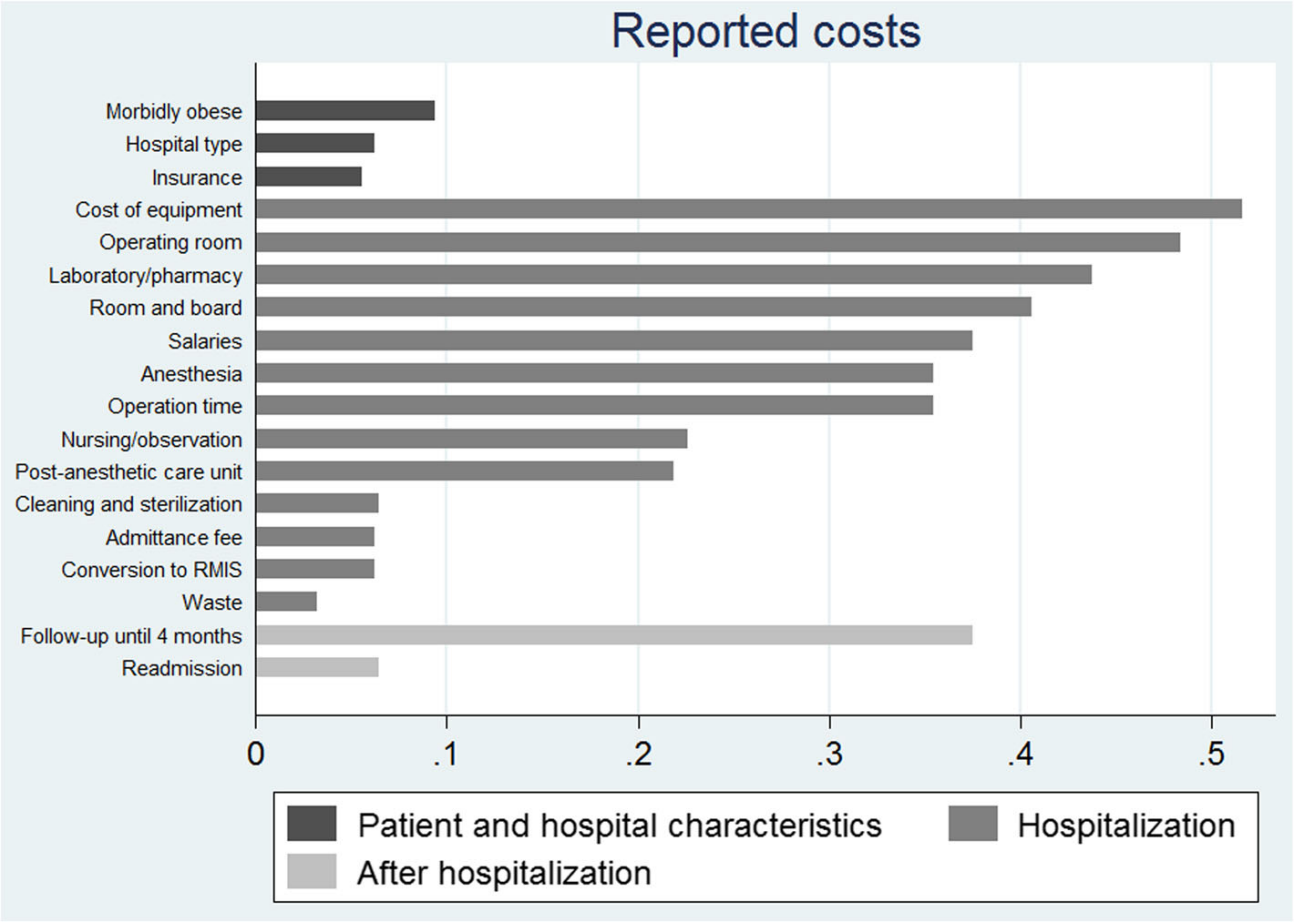
Reported costs from included studies

From the index hospitalization *Equipment costs* were the most common included cost items reported from the operation (*n* = 18) and the exact numbers of operation equipment costs were reported in six studies [9, 12, 14, 16, 17, 42]. Other costs included: costs of the operating room (*n* = 18), room and board (costs of regular room and any intensive care unit stay) (*n* = 14), other cost categories (e.g. pathology, laboratory, central supply, pharmacy, and infusion) (*n* = 14), operation time and costs of anesthesia (*n* = 13). Less frequently included costs were cleaning and sterilizing equipment (*n* = 4). Other reported costs not related to the index hospitalization were costs of the patient being morbidly obese and insurance coverage (*n* = 3). An overview of reported costs is presented in Fig. 2.

## Discussion

Our study showed that only few cost studies complied with the methodological items recommended by the CHEERS checklist. We identified critical design issues.. Ideally, a broader cost perspective is recommended to clarify resource use from hospitalization and outside the healthcare system. The time horizon should include resource consumption and costs following the intervention e.g. include rehabilitation.

Only few studies [11, 37, 43] included the societal perspective thus avoiding potentially important cost elements such as estimated costs related to the use of patient time e.g. return to labor market, transportation, and costs from the non-health care sector. Collecting costs from the hospital perspective, only short-term cost implications are considered. When the loss of productivity or other patient related use outside the hospital is not included; additional costs or savings of the surgical procedure will not be elucidated. The societal perspective includes both short- and long-term outcomes. If costs outside the hospital are not included, investments with high purchasing costs may seem less attractive.

The time horizon was reported in a few studies and most often costs from the inpatient stay and up to 4 months after surgery was given. With a short follow-up time after surgery cost drivers as rehabilitation and return to work are missing and all costs and effects from surgery are therefore not displayed [3, 44, 45].

Most of the studies used gross-costing based on data from records, chart reviews, or databases. However, when comparing RMIS with other surgical procedures, gross-costing lacks specificity and primarily defines a total budget instead of detailed data. In a pragmatic world, micro-costing is possible from a single hospital, whereas gross-costing data may be the only available data suitable to perform large-scale analyses. Micro-costing is desirable but may be very costly and time consuming to collect. If health care cost analyses have omitted clarity of which cost items they have included it may have a great impact on the transparency and hence the conclusions being used for decision making. Costs of operative procedures should be transparent and state which cost drivers are included and whether indirect costs such as cleaning and staff salary are included in the calculation. It may be difficult to rank the most important cost items in cost studies depending of the aim and data available. One of the most crucial cost items is the purchasing and maintenance costs of the robotic platform. This will affect the result and should be very clearly stated. The operation time may seem as an important cost item, where the exact time can influence the overall costs. When using charges, the overall costs are often based on an average of utensils, staff salary and operation time etc. for the specific operation and it is not possible to measure the smaller variations in time consumption. Even though robotic operations have shorter length of stay compared to open surgery, the taxes may seem unaffected. Hospitals are paid a fixed price for each patient treated.

The purchase of the robotic platform and the maintenance costs were rarely included although they represent an important opportunity costs and have a potential to display major impact on the cost analyses. An increased number of operations per year will decrease the cost per operation [2, 3] but only a few studies included the number of annual procedures or procedures performed per robotic platform. Of notice, Reynisson, P. et al. reported twice as high in-patient charge compared to other studies due to a minor depreciation time of the robot and a decreased number of yearly operations [18]. They also reported investment and equipment costs to be lower in North America than in Europe which should be taken into account when comparing the costs between countries [18].

Staff salaries may represent the most expensive cost driver when estimating the total costs. Only one study included staff salaries as a transparent cost estimation, dividing the time spent on the operation by the hourly wage [14]. A study by Lonnerfors, C. et al. defined major cost drivers influencing the hospital costs as duration of operating time and length of inpatient stay [17]. The majority of the studies did not report key cost items thus decreasing the generalizability.

Hospital charges based on what a health care provider bills for the service may be easily obtained from databases. However, hospital charges do not reflect the actual expenses; rather they represent local estimations of costs related to specific procedures. There can be great variations in the charges due to either over- or underestimation of the actual costs of hospital care, time variation, or variation in profit [32, 46]. Hence, use of the charging system may be inappropriate and prevent comparisons across countries or institutions due to variations in organizational structures, range of health care facilities, and different budgets.

Several studies have documented that great expertise with RMIS improves the surgical outcome in terms of decreased operation time, shorter length of stay and a decrease in readmissions [18, 26, 34, 38]. Many of the studies mentioned the importance of the learning curve of RMIS but did not adjust for it in the analysis. Laursen et al. examined the costs of RMIS in 7670 women over a period of 7 years showing costs to depend on the learning curve of RMIS [47]. Hence, although difficult to account for, the learning curve may represent an important cost driver.

## Conclusions

Inadequate reporting of the study perspective, short time horizon, and use of charge data decreased the methodological quality in studies of resource use and costs related to RMIS within gynecology. Few studies complied with the methodological items in the CHEERS checklist and the methodological quality was generally assessed low. If the results lack transparency of included cost elements it can lead to incorrect incentives for decision makers leading to inefficient allocation of hospital resources.
